# Biogenic Synthesis, Characterization, and Photocatalytic Evaluation of Pristine and Graphene-Loaded Zn_50_Mg_50_O Nanocomposites for Organic Dyes Removal

**DOI:** 10.3390/nano12162809

**Published:** 2022-08-16

**Authors:** Jayakaran Pachiyappan, Gnanasundaram Nirmala, Selvaraju Sivamani, Rajakumar Govindasamy, Muthu Thiruvengadam, Marina Derkho, Pavel Burkov, Aleksey Popovich, Vera Gribkova

**Affiliations:** 1 School of Chemical Engineering, Vellore Institute of Technology, Vellore 632014, Tamil Nadu, India; 2 Engineering Department, University of Technology and Applied Sciences, Salalah 211, Oman; 3 Department of Orthodontics, Saveetha Dental College and Hospitals, Saveetha Institute of Medical and Technical Sciences (SIMATS), Saveetha University, Chennai 600 077, Tamil Nadu, India; 4 Department of Applied Bioscience, College of Life and Environmental Science, Konkuk University, Seoul 05029, Korea; 5 Institute of Veterinary Medicine, South-Urals State Agrarian University, 13 Gagarin St., Troitsk, 457100 Chelyabinsk, Russia; 6 Department of Scientific Research, K.G. Razumovsky Moscow State University of Technologies and Management (The First Cossack University), 73 Zemlyanoy Val, 109004 Moscow, Russia

**Keywords:** red macroalgae, nanocomposites, photocatalysis, RSM

## Abstract

Algal biomass synthesised nanocomposites have a higher surface area and reusability advantages. This study aimed to synthesise and characterise ZnMgO and silica-supported graphene with ZnMgO (G-ZnMgO) nanocomposites from *Kappaphycusalvarezii* and evaluate their potential in the application of photocatalysis to remove Rhodamine-B (RhB) and methylene blue (MB) dyes from their aqueous medium by maximising the percentage removal using response surface methodology (RSM) modelling. Nanocomposites were synthesised and characterised by biogenic and instrumental (Powder X-ray diffraction (P-XRD), electron microscopic analysis (SEM and TEM), Fourier transform infrared spectroscopy (FTIR), Energy dispersive analysis of X-rays (EDAX). and UV-visible diffuse reflectance spectroscopy (UV-DRS)) methods, respectively; modelling predicted the optimal conditions to be photocatalyst dosage and contact time of 1 g/L and 90 min, respectively, to obtain maximum MB dye removal of 80% using G-ZnMgO. The results showed the best fit between experimental and RSM predicted values. Thus, the obtained results conclude that the algal biomass synthesised nanocomposites were found to be one of the potential photocatalysts for the removal of RhB and MB dyes from their aqueous solution.

## 1. Introduction

Unrefined dyes are predominant pollutants in wastewater treatment because they are frequently utilised in numerous industrialised applications and released into the ecosystem. The majority of chemicals are refractory, strong, coloring agents, or even poisonous and cancerous. Researchers have developed simple and efficient dye sewage treatment technologies as a consequence of the negative effects of dyes on public health and environments [1].

Even though widely used methods are available for wastewater treatment, photocatalysis has been studied for decades as a possible solution. It is also used for the synthesis of chemical compounds and energy-related advancement techniques. It allows the use of sunlight as a renewable and sustainable energy source. This method relies on the existence of microelectronics that could be activated by illumination of higher potency than its bandgap energy, producing pairs of electrons with a high level of energy that can be employed in electrochemical reactions to degrade dyes. Photocatalysis is often used for the activation of a unit process by a solid (photocatalyst) irradiated by a light (UV/visible) source. Due to their great photosensitivity and stability, semiconductor metal oxide nanocomposites have been used as photocatalysts [2]. Titanium dioxide (TiO_2_) is the most extensively utilised photocatalyst for the breakdown of organic contaminants among the many semiconductor materials. However, TiO_2_ requires solar light for efficient photocatalysis [3].

In the visible-light photocatalytic area, ZnO and MgO are potential low-cost and narrow bandgap materials. Zn-doped MgO (ZnMgO) can absorb the most visible light. The formation of nano-sized ZnMgO particles can be accomplished in a number of approaches, including the sol-gel technique, chemically gaseous phase deposition, sensor evaporation, hydrothermal method, and combustible aerosols production, in accordance with the research. Green synthesis of ZnMgO using seaweed has been reported [4], but it has not been widely exploited yet.

Response surface methodology develops a quadratic model with minimum trials and errors. Additionally, it is used to study the interaction between independent factors [5]. It has two designs: Box–Wilson and Box–Behnken design. According to their principle, the number of trials required to conduct for the Box–Wilson design is 2^f^ + 2*f + m and 2*f*(f−1) + m for the Box–Behnken design, where f is the quantity of individual factors and m is the quantity of midpoint [6]. Normally, the Box–Wilson design requires more experiments and produces more accurate results than the Box–Behnken design. Box–Wilson design starts with analysis for a minimum of two independent factors, whereas the Box–Behnken design starts with a minimum of three factors.

In this work, Zn_50_Mg_50_O and G-Zn_50_Mg_50_O (silica-supported graphene with Zn_50_Mg_50_O) nanocomposites were synthesised using red algae *Kappaphycus alvarezii* as a stabilising agent by co-precipitation method to improve the ultraviolet receptive capability of metallic photo-catalysts (doping with other elements) by altering the physical and chemical properties of the metal oxides used in this study. The properties of nanocomposite were characterised by powder X-ray diffraction (P-XRD), UV-visible diffuse reflectance spectroscopy (UV-DRS), scanning electron microscopy (SEM) along with energy dispersive analysis of X-rays (EDAX), Transmission electron microscope (TEM), and Fourier transform infrared spectroscopy (FTIR) for their crystallinity, surface morphology, elemental composition, optical direct band gap energy, and functional groups respectively. The photocatalytic activities of nanocomposites (Zn_50_Mg_50_O and G-Zn_50_Mg_50_O) were studied under visible light for the decomposition of RhB and MB dye molecules.

## 2. Materials and Methods

### 2.1. Chemicals

All the chemicals utilised in this investigation were of the highest purity and analytical grade. Sugarcane juice was bought at a nearby market. Silica gel, Rhodamine (RhB), Methylene Blue (MB), Zinc acetate (Zn(CH₃CO_2_)_2_), magnesium nitrate (Mg(NO_3_)_2_), Sodium hydroxide (NaOH), Sodium sulfate, and Triton X-100 were purchased from Sigma Aldrich (St. Louis, MO, USA). The overall study was performed with Milli-Q water. An electronic balance was used to weigh each chemical (Shimadzu, Kyoto, Japan).

### 2.2. Collection and Preparation of Algae Extract

*Kappaphycus alvarezii*, marine red macroalgae, was collected in Mandapam, Rameswaram, Tamil Nadu, India. The algal product was often rinsed, dehydrated, and pulverised before being used. A total of 5 g of sample was mixed with 500 mL of Milli-Q water and soaked overnight. The aqueous filtrate was collected. Furthermore, the filtrate was utilised to produce nanocomposite, Zn_50_Mg_50_O.

### 2.3. Synthesis of Zn_50_Mg_50_O and Silica-Supported Graphene with Zn_50_Mg_50_O Nanocomposites

The ZnMgO nanocomposite (Zn_50_Mg_50_O) was prepared by using 0.1 M of molar ratio of substrates (Zn(CH₃CO_2_)_2_: Mg(NO_3_)_2_, 1:4), the solution with the addition of *Kappaphycus alvarezii* extract and sodium hydroxide (NaOH-0.2 M) acting as the reducing agent. It is designated as Zn_50_Mg_50_O. Concentrated sugarcane juice (100 mL) was mixed with 200 g of silica gel and added with 300 g of prepared Zn_50_Mg_50_O nanocomposite, and the mixture was dried at 100 °C for 1 h. After drying the composite, calcination was continued for 3 h at 350 °C in N_2_ atmosphere in a furnace. It is designated as G-Zn_50_Mg_50_O.

### 2.4. Characterisation of Nanocomposites

The characteristics of nanoparticles and nanocomposites were characterised by P-XRD, UV-visible diffuse reflectance spectroscopy (UV-DRS), electron microscopic analysis (SEM), EDAX, and Fourier transform infrared spectroscopy (FTIR) for their crystallinity, surface morphology, elemental composition, optical direct band gap energy, and functional groups, respectively.

### 2.5. Batch Photocatalytic Studies

Nanocomposites (Zn_50_Mg_50_O and G-Zn_50_Mg_50_O) were utilised for the degradation of RhB and MB dyes using UV illumination (100 W Tungsten halogen lamp). A measured dosage of catalytic material was applied to 75 mL of dye (RhB or MB) solution in a standard experimental setup. The suspension was extensively stirred in the dark before light irradiation to create an adsorption–desorption equilibrium of dye onto the catalyst. Three mL of aliquot was obtained at regular intervals during the procedure and filtered to eliminate the catalyst particles. A UV spectrophotometer was used to calculate the absorption of RhB and MB at regular intervals at their distinctive absorption wavelengths of 554 and 664 nm, respectively. The illumination was from a 100 W halogen lamp, and the electrolyte solution was an aqueous 0.1 M Na_2_SO_4_ solution. The working electrode prepared using 10 mg of the prepared nanomaterials were combined with Triton X-100 (20 µL) and purified water (40 µL) to create a slurry, which was then coated on an FTO glass substrate using the doctor blade technique. The electrode’s active surface area was 0.5 × 0.5 cm^2^, and the sticky tape served as a separator for a triple-layered homogeneous coating. It was then dried for 3 h at 120 °C, following the method of Theerthagiri et al. [7].

### 2.6. Response Surface Methodology

The surface response modelling and analysis were carried out using Stat-Ease, Inc.’s original Design-Expert 13 programme. In this study, the Box–Wilson technique was chosen to optimise the amount of dye removal at the appropriate photocatalyst dosage and contact time. All investigations were carried out in accordance with the Box–Wilson parametric study, and the significance of the experimental data’s fit to linear, 2FI (two-factor interaction), quadratic, and cubic models was investigated. The variables were again matched to the important quadratic formula to analyse the impact of independent factors on the response, which is depicted in Formula (1), and model coefficients were assessed using Formula (2). The coefficients were then evaluated to examine the difference among experimental and simulation results in Equation (1). Analysis of variance (ANOVA) was used to determine the model’s significance using the highest F-value, lowest *p*-value, and a 95% confidence level. The goodness-of-fit between predicted and experimental values was also evaluated using the regression coefficient R^2^, the difference between modified and projected R^2^, and predicted residual error sum of squares (PRESS) values.

(1) $$Y=\beta_{0}+\overset{n}{\underset{i=1}{\sum }}\beta_{i}X_{i}+\overset{n}{\underset{i=1}{\sum }}\beta_{ii}X_{i}^{2}+\overset{n-1}{\underset{i=1}{\sum }}\overset{n}{\underset{j=i+1}{\sum }}\beta_{ij}X_{i}X_{j}+\varepsilon$$

(2) $$\beta ={\left(X^{T}X\right)}^{-1}\left(X^{T}Y\right)$$

where: *β*_0_ is an endpoint, *β_i_*, *β_ii_*, *β_ij_* are quadratic, square, and interactions factors, *Y* is the response, respectively, *X_i_* and *X_j_* are significant indicators, and *ε* is a random variable.

## 3. Results and Discussion

### 3.1. Powder XRD Analysis

**Figure 1:**
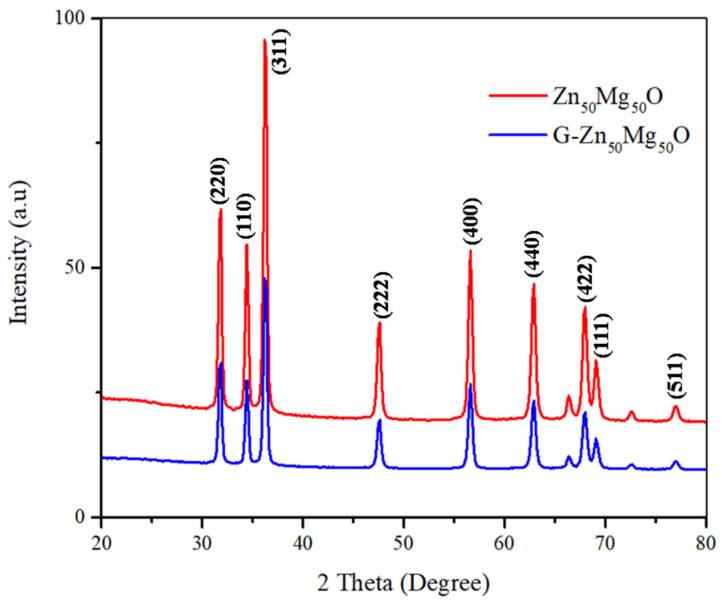
XRD spectra of Zn_50_Mg_50_O and G-Zn_50_Mg_50_O.

Zn_50_Mg_50_O and G-Zn_50_Mg_50_O nanocomposites were analyzed for their crystallinity using powder X-ray diffractometry (P-XRD), as shown in Figure 1. Nanocomposites’ diffraction patterns were evaluated according to the simultaneous analyses shown in JCPDS Nos. 01-079-0207 and.00-004-0821, respectively, and are closely aligned with the reported 2θ values. CuKα_1_ radiation (λ = 1.5406 Å), 40 kV–40 mA, 2θ scanning technique was used to analyze the X-ray diffraction peaks of prepared nanocomposites samples. The data were collected between 20 and 80 degrees. The diffraction patterns were detected using the miller indexes (h,k,l), and the resulting P-XRD spectra revealed that the prepared nanocomposites were all nanocrystals in nature. The sharp peaks of P-XRD demonstrate the reactive component of MgO and ZnO. The actual percentage proportion of the nanomaterials corresponded with data peaks by P-XRD when there were less impurities. The nanocomposites’ purity and crystalline size were determined to be excellent [8,9].

### 3.2. UV-DRS

**Figure 2:**
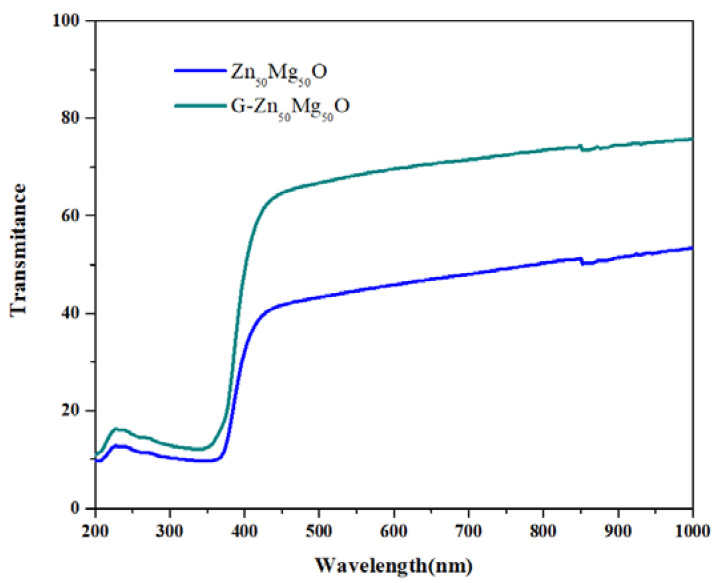
UV-DR spectra of Zn_50_Mg_50_O and G-Zn_50_Mg_50_O.

Applying the Tauc relationship [10] given in Equation (3), the optic band gap energy (*E_g_*) of the biogenic produced nanocomposites was determined.

(3) $$\alpha h\nu =A\;{\left(h\nu -E_{g}\right)}^{n}$$

where *hν*—the photon energy, *α*—the absorption co-efficient, *A*—constant and *n* = ½ for direct transition and *n* = 2 for indirect transition [11]. If the velocity of the conduction band’s electrons and electron holes are equal, an electron will emit a photon in the straight transfer. The direct band difference energy is equivalent to the photon’s energy [12,13]. A plot between *(αhυ)*^2^ and *hυ* is drawn in order to derive the photonic direct band gap energies from the UV-DRS absorption spectra. Figure 2′s straight-line extrapolation to the *(αhν)*^2^ = 0 axis yields the optical direct band gap energy value. The predicted optical direct band gap energies of green synthesised Zn_50_Mg_50_O and G-Zn_50_Mg_50_O were 3.08 and 4.71 eV, respectively. The energy gap for semiconductors lies between conductors and insulators. The visible light absorption capacity decreased as the graphene addition increased.

### 3.3. SEM-EDAX

**Figure 3:**
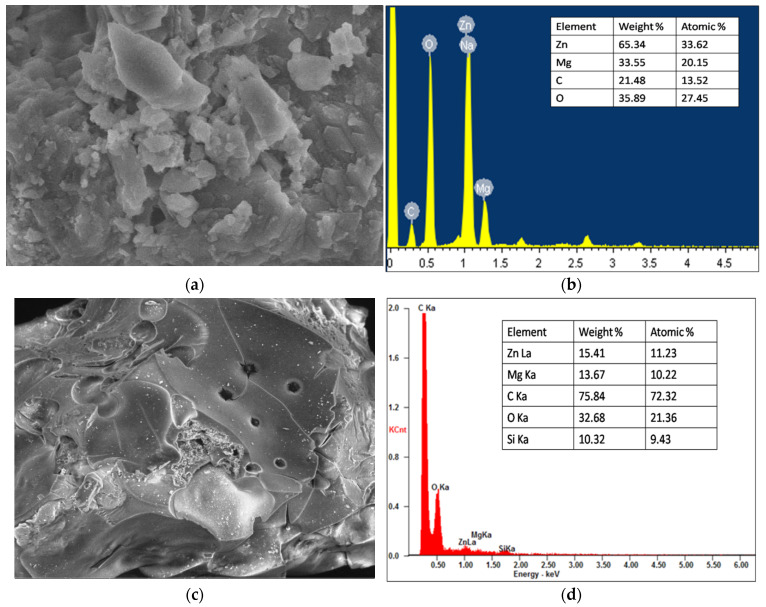
SEM images of (**a**) Zn_50_Mg_50_O and (**c**) G-Zn_50_Mg_50_O and EDAX spectra of (**b**) Zn_50_Mg_50_O and (**d**) G-Zn_50_Mg_50_O.

HR-SEM images of Zn_50_Mg_50_O and G-Zn_50_Mg_50_O nanocomposite materials are shown in Figure 3a–d, which clearly illustrate the surface characteristics of nanomaterials synthesised by the biogenic co-precipitation technique. ZnO NPs were rod-shaped, with diameters ranging from 57 to 68 nm, as seen in Figure 3a. EDAX findings revealed the existence of major elements: Zn, Mg, and O (Figure 3b) [14,15]. Figure 3c shows the surface morphology of G-Zn_50_Mg_50_O nanocomposite.

In Figure 3c, MgO and ZnO particles were randomly coated on the surface of graphene, and micrographs revealed a trace content of ZnO nanorods and particles with hexagonal MgO nanopellets. The large accumulation of particles on the surface of graphene is due to the possibility of carbon-based bonds forming during the synthesis. Notably, the addition of ZnO and graphene, as well as thermal processes during nanocomposites synthesis, had no impact on the morphological properties of MgO. The EDAX spectra analysis was used to evaluate the degree of distribution of various elements on the nanocomposite surface (Figure 3d), which revealed a standardised distribution of major elements (Mg, O, Zn, Si, and C) on the catalyst surface. This discovery revealed that the synthesis process was completed successfully, with no ashes or impurities present in the samples.

### 3.4. TEM

**Figure 4:**
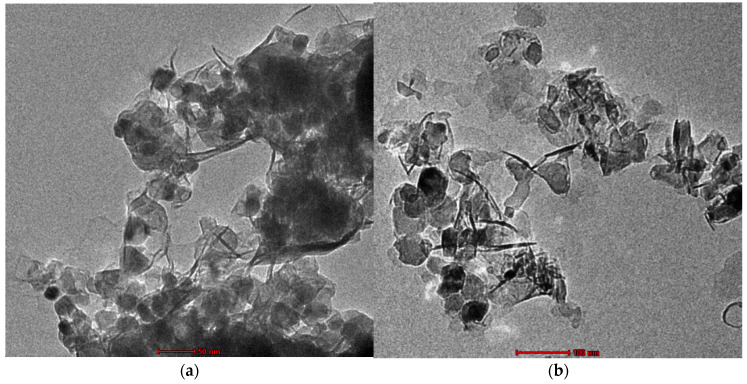
TEM images of (**a**) Zn_50_Mg_50_O and (**b**) G-Zn_50_Mg_50_O.

Transmission Electron Microscope (TEM) analysis is a simple as well as an effective way to examine the morphology of nanoparticles and nanocomposites. Figure 4a,b shows the structural and morphological characteristics of Zn_50_Mg_50_O and G-Zn_50_Mg_50_O nanocomposites, which display hexagonal, rod, and irregular morphology with sizes of 55–70 and 60–70 nm, respectively [16,17].

### 3.5. FTIR

**Figure 5:**
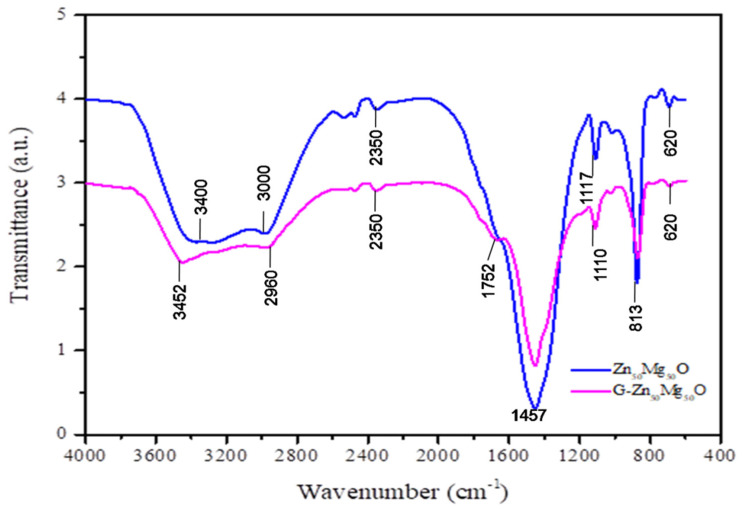
FTIR spectra of Zn_50_Mg_50_O and G-Zn_50_Mg_50_O.

FTIR analysis showed a broad peak at 3452 cm^−1^ and 2960 cm^−1^ due to the major alcoholic O-H functional group, which is the important shifted metal ion absorption point [13,18,19,20,21]. Peaks were produced by the excitations of -CN and CH_3_ at 1752 cm^−1^ and 1457 cm^−1^, respectively (Figure 5). The peak at 1187 cm^−1^ could be shifted by the OH bond’s bending vibration, which is associated with the water surface that was absorbed by the synthetic ZnO/MgO nanocomposites. The Mg–O and Zn–O bindings that serve to shape pure and composite types of synthetic metal oxides may be responsible for the peaks at 813 cm^−1^ and 620 cm^−1^.

### 3.6. RSM Analysis for Photocatalytic Studies

The independent variables and values used for the photocatalytic removal of dyes using nanocomposites are shown in Table 1. Independent variables and quantities were chosen from early experimental investigation and a review of the literature. In the Box–Wilson design, there are (2^f^ + 2*f + m) experiments, where f is the number of independent numeric factors and m is the number of mid-point. Hence, the number of experiments carried out was (2^2^ + 2*2 + 3) = 11. The Box–Wilson matrix of 11 trials for photocatalytic dye removal using nanocomposites is shown in Table 2.

The numerous models, linear, 2FI, quadratic, and cubic models, were analyzed for photocatalytic removal of dyes using nanocomposites. The values of *F*, *p*, R^2^, and the difference between modified and expected R^2^ and PRESS were utilised to verify the significance of the model. A high *F*-value of 56.75, a low *p*-value of <0.05, R^2^ > 0.8, the difference between modified and expected R^2^ < 0.2, and the least value of PRESS implied the important model and suitable for photocatalytic removal. The model fitting technique revealed that the quadratic equation fitted well with the investigational data of photocatalytic degradation of dyes using nanocomposites.

Equations (4)–(7) represent the results of fitting the experimental data to the quadratic model.

(4) $$Y=-8.241+75.68A+0.643B+0.042AB-44.74A^{2}-0.001B^{2}$$

(5) $$Y=-5.941+74.85A+0.634B+0.042AB-44.74A^{2}-0.001B^{2}$$

(6) $$Y=-0.969+63.52A+0.701B+0.016AB-35.16A^{2}-0.001B^{2}$$

(7) $$Y=4.679+47.51A+0.748B+0.033AB-22.93A^{2}-0.002B^{2}\;$$

The quadratic models describing the photocatalytic degradation of RhB and MB dyes using Zn_50_Mg_50_O are represented by Equations (4) and (5), respectively. The quadratic models describing the photocatalytic degradation of RhB and MB dyes using G-Zn_50_Mg_50_O nanocomposite are represented by Equations (6) and (7), respectively. The reaction generally decreased when negative coefficient terms increased and vice versa. In a similar manner, a decrease in positive coefficient terms tended to reduce the dependent factor and vice versa [22]. According to the maxima and minima principle, positive coefficient values in linear terms and negative coefficient values in quadratic terms tend to maximise the response. Equations (3)–(6) thus showed that the linear and quadratic terms’ positive and negative coefficients tended to optimise dye removal.

Table 3 show the analysis of variance (ANOVA) for the quadratic model developed for photocatalytic removal of dyes using nanocomposites. From the ANOVA table, it is evident that the model was significant, with *F*-values ranging between 80.19 and 188.68 and a low *p*-value of <0.05. For photocatalytic degradation of RhB and MB dyes using Zn_50_Mg_50_O, the interaction between photocatalyst dosage and time was insignificant, with a *p*-value > 0.05. Linear terms for photocatalytic removal were significant with a *p*-value < 0.05. This means that the linear terms have more effect on dye removal. For photocatalytic degradation of RhB and MB dyes using Zn_50_Mg_50_O, quadratic terms were significant with a *p*-value > 0.05. For photocatalytic degradation of RhB and MB dyes using G-Zn_50_Mg_50_O, the quadratic term on time was significant with a *p*-value > 0.05.

**Figure 6:**
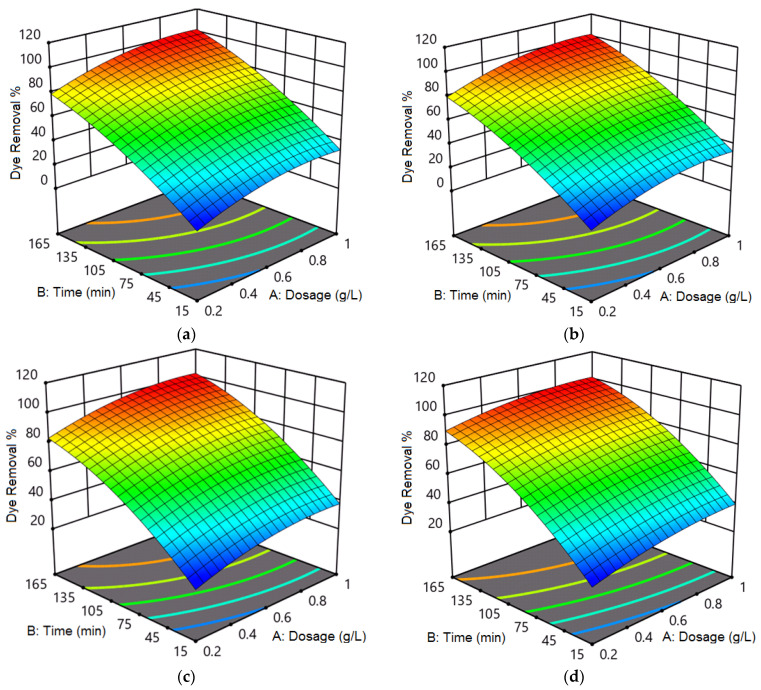
Interactive effect of photocatalyst dosage and contact time on photocatalytic removal of (**a**) RhB and (**b**) MB dyes using Zn_50_Mg_50_O, and (**c**) RhB and (**d**) MB dyes using G-Zn_50_Mg_50_O.

Figure 6 shows the interactive effect of photocatalyst dosage and contact time on photocatalytic removal of (a) RhB and (b) MB dyes using Zn_50_Mg_50_O, and (c) RhB and (d) MB dyes using G-Zn_50_Mg_50_O. From Figure 6a, the photocatalytic removal of 17.7% was obtained for RhB dye using Zn_50_Mg_50_O at the lowest photocatalyst dosage and contact time of 0.2 g/L and 15 min, respectively. At this point, when the photocatalyst dosage increased to 0.6 g/L, the dye removal enhanced to 26.2%. Further increment of photocatalyst dosage to 1 g/L led to an increase in dye removal to 34.2%. Similarly, the dye removal increased to 51% at the photocatalyst dosage and contact time of 0.2 g/L and 90 min, respectively. The dye removal increased to 78.5% when the contact time increased to 165 min at the same photocatalyst dosage of 0.2 g/L. Additionally, the dye removals of 72% and 100% were obtained at a contact time of 90 and 165 min at the photocatalyst dosage of 0.6 g/L. Finally, the dye removals of 74.8% and 100% were observed at contact time values of 90 and 165 min at the photocatalyst dosage of 1 g/L. From the interactive effect between photocatalyst dosage and contact time, the optimal conditions were found to be photocatalyst dosage and contact time of 0.6 g/L and 165 min, respectively, to obtain dye removal of 100%.

From Figure 6b, the photocatalytic removal of 24.7% was obtained for MB dye using Zn_50_Mg_50_O at the lowest photocatalyst dosage and contact time of 0.2 g/L and 15 min, respectively. At this point, when the photocatalyst dosage increased to 0.6 g/L, the dye removal enhanced to 29.2%. Further increment of photocatalyst dosage to 1 g/L led to an increase in dye removal to 39.2%. Similarly, the dye removal increased to 56.1% at the photocatalyst dosage and contact time of 0.2 g/L and 90 min, respectively. The dye removal increased to 83.6% when the contact time increased to 165 min at the same photocatalyst dosage of 0.2 g/L. Additionally, the dye removals of 76.7% and 100% were obtained at a contact time of 90 and 165 min at the photocatalyst dosage of 0.6 g/L. Finally, the dye removals of 79.8% and 100% were observed at contact time values of 90 and 165 min at the photocatalyst dosage of 1 g/L. From the interactive effect between photocatalyst dosage and contact time, the optimal conditions were found to be photocatalyst dosage and contact time of 0.6 g/L and 165 min, respectively, to obtain dye removal of 100%.

From Figure 6c, the photocatalytic removal of 19.7% was obtained for RhB dye using G-Zn_50_Mg_50_O at the lowest photocatalyst dosage and contact time of 0.2 g/L and 15 min, respectively. At this point, when the photocatalyst dosage increased to 0.6 g/L, the dye removal enhanced to 28.2%. Further increment of photocatalyst dosage to 1 g/L led to an increase in dye removal to 35.2%. Similarly, the dye removal increased to 52% at the photocatalyst dosage and contact time of 0.2 g/L and 90 min, respectively. The dye removal increased to 79.5% when the contact time increased to 165 min at the same photocatalyst dosage of 0.2 g/L. Additionally, the dye removals of 72.9% and 100% were obtained at a contact time of 90 and 165 min at the photocatalyst dosage of 0.6 g/L. Finally, the dye removals of 75.8% and 100% were observed at contact time values of 90 and 165 min at the photocatalyst dosage of 1 g/L. From the interactive effect between photocatalyst dosage and contact time, the optimal conditions were found to be photocatalyst dosage and contact time of 0.6 g/L and 165 min, respectively, to obtain dye removal of 100%.

From Figure 6d, the photocatalytic removal of 26.7% was obtained for MB dye using G-Zn_50_Mg_50_O at the lowest photocatalyst dosage and contact time of 0.2 g/L and 15 min, respectively. At this point, when the photocatalyst dosage increased to 0.6 g/L, the dye removal enhanced to 32.2%. Further increment of photocatalyst dosage to 1 g/L led to an increase in dye removal to 40.2%. Similarly, the dye removal increased to 63% at the photocatalyst dosage and contact time of 0.2 g/L and 90 min, respectively. The dye removal increased to 90.5% when the contact time increased to 165 min at the same photocatalyst dosage of 0.2 g/L. Additionally, the dye removals of 77.8% and 100% were obtained at a contact time of 90 and 165 min at the photocatalyst dosage of 0.6 g/L. Finally, the dye removals of 80.8% and 100% were observed at contact time values of 90 and 165 min at the photocatalyst dosage of 1 g/L. From the interactive effect between photocatalyst dosage and contact time, the optimal conditions were found to be photocatalyst dosage and contact time of 0.6 g/L and 165 min, respectively, to obtain dye removal of 100%.

Experiments were conducted under different production settings given in Table 2 to evaluate the validity of the proposed model and the resulting predicted results. The deviation between the investigational and expected values was 3.43, which is lesser than ±5%. The values obtained were not significantly different from the expected values, according to the coefficient of variation (CV), which was 6.56%. Additionally, it was demonstrated that the difference between observed and expected values is small. The analysis of the data was supported by the fact that all of the data points were within 10% of the expected and experimental values.

## 4. Conclusions

The current investigation aimed to prepare and investigate ZnMgO and silica-supported graphene with ZnMgO (G-ZnMgO) nanocomposites and evaluate their potential in the application of photocatalysis to remove RhB and MB dyes from their *Kappaphycus alvarezii* aqueous extract by maximising the percentage removal using response surface methodology (RSM) modelling. RSM modelling predicted the optimal conditions were found to be photocatalyst dosage and contact time of 0.56 g/L and 165 min, respectively, to obtain maximum MB dye removal of 10% using G-Zn_50_Mg_50_O. For RhB using Zn_50_Mg_50_O and G-Zn_50_Mg_50_Oand MB using Zn_50_Mg_50_O, 100% removal was achieved at photocatalyst dosage between 0.58 and 0.62 g/L and 165 min. The results showed the best fit between experimental and RSM predicted values. Thus, the obtained results conclude that the algal biomass synthesised nanocomposites were found to be one of the potential photocatalysts for the degradation of RhB and MB dyes from *Kappaphycus alvarezii* aqueous solution.

## Figures and Tables

**Table 1:** Independent variables and quantities utilised for photocatalytic removal of dyes using nanocomposites.

Independent Factor	Symbol	Unit	Levels		
			Low (−1)	Centre (0)	High (+1)
Photocatalyst dosage	A	g/L	0.2	0.6	1
Contact time	B	min	15	90	165

**Table 2:** Box–Wilson design matrix for photocatalytic removal of dyes using nanocomposites.

Std	Run	A: Photocatalyst Dosage (g/L)	B: Contact Time (min)	Dye Removal (%)			
				RhB—Zn_50_Mg_50_O	MB—Zn_50_Mg_50_O	RhB—G-Zn_50_Mg_50_O	MB—G-Zn_50_Mg_50_O
2	1	0.2	165	78.5	83.6	79.5	90.5
8	2	0.2	15	17.7	24.7	19.7	26.7
5	3	1	15	34.2	39.2	35.2	40.2
4	4	0.6	165	100	100	100	100
11	5	0.6	15	26.2	29.2	28.2	32.2
9	6	0.6	90	71.8	76.9	72.8	77.7
7	7	0.6	90	72	76.7	72.9	77.8
6	8	1	90	74.8	79.8	75.8	80.8
3	9	0.6	90	71.9	76.8	73	77.6
1	10	0.2	90	51	56.1	52	63
10	11	1	165	100	100	100	100

**Table 3:** Analysis of variance (ANOVA) for the quadratic model developed for photocatalytic dye removal.

**(a) RhB dye using Zn_50_Mg_50_O composite**					
**Source**	**Sum of Squares**	**Df**	**Mean Square**	**F-Value**	***p*** **-Value**
Model	7680.36	5	1536.07	147.17	<0.0001
A-Photocatalyst dosage	636.54	1	636.54	60.99	0.0006
B-Time	6693.36	1	6693.36	641.29	<0.0001
AB	6.25	1	6.25	0.5988	0.4740
A²	129.80	1	129.80	12.44	0.0168
B²	122.64	1	122.64	11.75	0.0187
Residual	52.19	5	10.44		
Lack of Fit	52.17	3	17.39	1738.89	
Pure Error	0.0200	2	0.0100		
Cor Total	7732.55	10			
**(b) MB dye using Zn_50_Mg_50_O composite**					
**Source**	**Sum of Squares**	**df**	**Mean Square**	**F-Value**	***p*** **-Value**
Model	7375.29	5	1475.06	150.35	<0.0001
A-Photocatalyst dosage	596.01	1	596.01	60.75	0.0006
B-Time	6428.83	1	6428.83	655.29	<0.0001
AB	6.25	1	6.25	0.6371	0.4610
A²	129.80	1	129.80	13.23	0.0149
B²	122.64	1	122.64	12.50	0.0166
Residual	49.05	5	9.81		
Lack of Fit	49.03	3	16.34	1634.45	
Pure Error	0.0200	2	0.0100		
Cor Total	7424.35	10			
**(c) RhB dye using G-Zn_50_Mg_50_O composite**					
**Source**	**Sum of Squares**	**df**	**Mean Square**	**F-Value**	***p*** **-Value**
Model	6925.68	5	1385.14	80.19	<0.0001
A-Photocatalyst dosage	496.86	1	496.86	28.76	0.0030
B-Time	6048.37	1	6048.37	350.15	<0.0001
AB	0.9025	1	0.9025	0.0522	0.8283
A²	80.19	1	80.19	4.64	0.0838
B²	204.12	1	204.12	11.82	0.0185
Residual	86.37	5	17.27		
Lack of Fit	86.35	3	28.78	2878.30	
Pure Error	0.0200	2	0.0100		
Cor Total	7012.05	10			
**(d) MB dye using G-Zn_50_Mg_50_O composite**					
**Source**	**Sum of Squares**	**df**	**Mean Square**	**F-Value**	***p*** **-Value**
Model	6718.83	5	1343.77	188.68	<0.0001
A-Photocatalyst dosage	277.44	1	277.44	38.96	0.0015
B-Time	6105.66	1	6105.66	857.31	<0.0001
AB	4.00	1	4.00	0.5616	0.4873
A²	34.09	1	34.09	4.79	0.0803
B²	227.12	1	227.12	31.89	0.0024
Residual	35.61	5	7.12		
Lack of Fit	35.59	3	11.86	1186.32	
Pure Error	0.0200	2	0.0100		
Cor Total	6754.44	10			

## Data Availability

Not applicable.
